# Evolving FATE: A New Lens on the Pathogenesis and Management of Feline Cardiogenic Arterial Thromboembolism

**DOI:** 10.3390/ani15111630

**Published:** 2025-06-01

**Authors:** Natasha S. Yeh, Meg Shaverdian, Ronald H. L. Li

**Affiliations:** 1 Terry Veterinary Medical Center, College of Veterinary Medicine, North Carolina State University, Raleigh, NC 27606, USA; nyeh@ncsu.edu; 2 Department of Surgical and Radiological Sciences, School of Veterinary Medicine, University of California, Davis, CA 95616, USA; mshaverdian@ucdavis.edu; 3 Department of Clinical Sciences, College of Veterinary Medicine, North Carolina State University, Raleigh, NC 27606, USA

**Keywords:** clopidogrel resistance, point of care testing, echocardiogram, saddle thrombus

## Abstract

Feline cardiogenic arterial thromboembolism (FATE) remains one of the most devastating complications of feline cardiomyopathies, with high mortality and recurrence rates. Despite its clinical importance, significant knowledge gaps persist in our understanding of FATE’s pathogenesis and optimal management strategies. Our review aims to address these gaps by providing a comprehensive overview of the current understanding of FATE, including disease mechanisms, risk factors, emerging diagnostics, and preventative strategies. Importantly, we identify key areas such as immunothrombosis, procoagulant platelets, platelet heterogeneity, and altered fibrinolysis where future research may yield novel biomarkers and therapeutic targets to improve outcomes in affected feline patients.

## Simple Summary

Feline arterial thromboembolism (FATE) is a severe and often fatal complication of feline cardiomyopathy, associated with a poor prognosis. Despite its clinical significance, the underlying pathophysiology remains incompletely understood. Current management strategies primarily focus on secondary prevention after a thromboembolic event, with limited emphasis on mitigating risk before onset. This review highlights emerging areas of research, including the roles of immunothrombosis, platelet heterogeneity, genetic testing, and precision medicine. Advancements in these fields may support the development of improved diagnostics and targeted therapies, ultimately enhancing outcomes for affected feline patients.

## 1. Introduction

**Figure 1:**
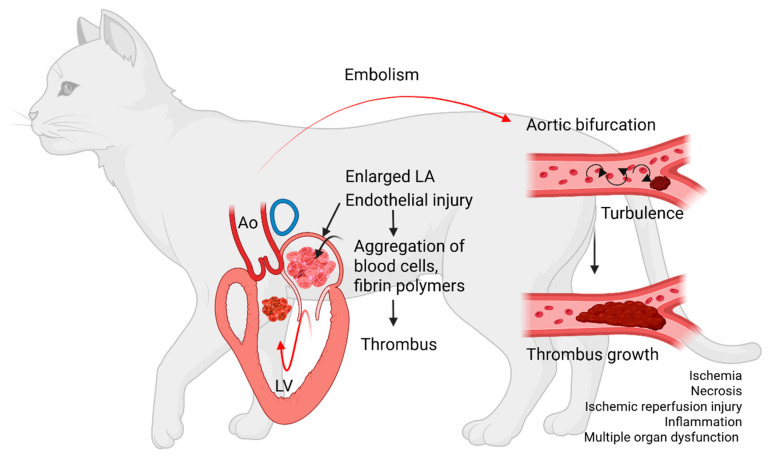
Diagram summarizing the sequence of events leading to cardiogenic arterial thromboembolism. Aggregates of blood cells and fibrin polymers settle to form an intracardiac thrombus inside a diseased and enlarged left atrium (LA). The thrombus then dislodges, travels through the left ventricle (LV) and aorta (Ao) to embolize to distal arteries. The thrombus embolizes most commonly to the aortic bifurcation, causing partial or complete obstruction. Stenosis of the vascular lumen creates blood flow turbulence, which facilitates further thrombus growth. Arterial thrombosis leads to impedance of blood flow, tissue ischemia, necrosis, ischemic reperfusion injuries, and multiple organ failure.

Feline cardiogenic arterial thromboembolism (FATE) is a devastating complication in cats with cardiomyopathies. FATE occurs when an intracardiac thrombus develops, dislodges into the systemic circulation, and embolizes to distal arteries, where partial or complete obstruction of blood flow in the systemic vasculature impedes blood flow, creating blood turbulence and thrombus growth. This leads to tissue ischemia, pain, tissue necrosis, hypoperfusion, ischemic-reperfusion injury, and systemic inflammation (Figure 1) [1]. The mortality rate reported is as high as 54%, with nearly one-third of cats euthanized shortly after diagnosis, and about 50% of cats died within 48 h of initial hospitalization. Long-term prognosis in cats that survived beyond hospital discharge is variable, with a recurrence of FATE ranging from 16.7% to 75% [2,3,4,5,6]. However, this is greatly influenced by the antithrombotic strategy for a FATE event. Other factors that contribute to poor outcomes include concurrent congestive heart failure (CHF), atrial fibrillation, and sudden death [1,3,5,7,8,9]. Due to the poor prognosis of FATE, primary prevention in high-risk patients is crucial to reducing the risk of intracardiac thrombosis and subsequent thromboembolic events. However, effective prevention depends on the timely recognition of risk factors; yet veterinarians currently have limited tools to reliably identify predisposed cats. Current diagnostic measures rely solely on echocardiographic assessments; however, this is complicated by the fact that most at-risk cats are clinically asymptomatic and lack auscultatory abnormalities that would typically indicate the need for echocardiographic evaluation. In addition, current evidence in antithrombotic strategies heavily emphasizes secondary prevention following an initial FATE event, while largely neglecting primary prevention [1,3,6]. Moreover, existing diagnostic and therapeutic approaches do not consider the contribution of the immune system in driving immunothrombosis and systemic hypercoagulability. This review aims to provide a brief overview of the current understanding and knowledge gaps in FATE pathophysiology, risk assessment, and antithrombotic strategies. Additionally, we will propose and highlight some future research directions to bridge these gaps, ultimately advancing knowledge and improving outcomes for cats with FATE.

## 2. A Review of FATE Pathophysiology: Current Evidence and Knowledge Gap

**Figure 2:**
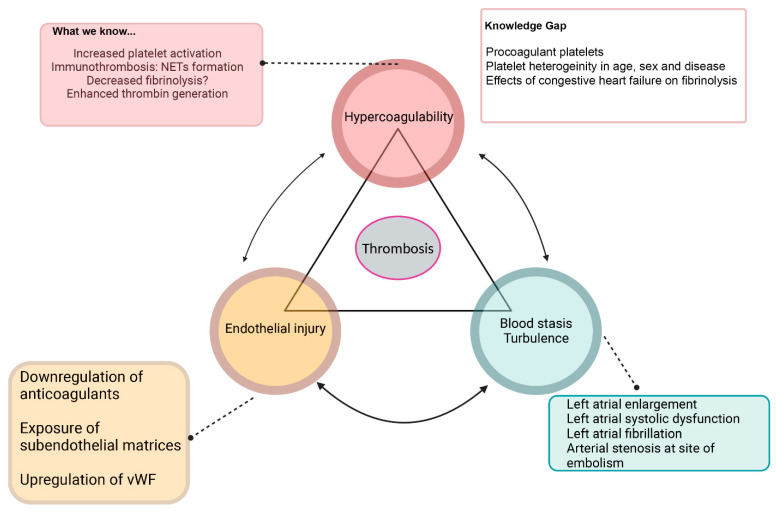
Summary of Virchow’s triad and known causes and knowledge gaps of derangements of each component, which includes systemic hypercoagulability, blood flow stasis, turbulence, and endothelial injury. NETs—neutrophil extracellular traps, vWF—von Willebrand factor.

Hemostatic balance is maintained by several regulatory mechanisms. In healthy animals, anticoagulant factors are slightly favored to maintain blood as a fluid state [10]. However, upon vascular injuries, primary and secondary hemostasis are activated to induce clot formation and wound healing [10]. While FATE pathophysiology is complex and not well understood, cardiomyopathies in cats can impact each of the three components of Virchow’s triad. Figure 2 summarizes the current understanding and knowledge gaps in the pathogenesis of FATE based on Virchow’s triad [1,8].

### 2.1. Platelet Activation

Systemic hypercoagulability as a leading cause of FATE has been explored. Platelets, the principal effector cells of primary hemostasis, are responsible for forming the initial platelet plug via the three-stage model of platelet activation, which involves adhesion, activation, and stabilization [9]. To date, a few ex vivo studies have documented increased platelet activation in cats with subclinical and clinical hypertrophic cardiomyopathy (HCM), which is the most prevalent form of cardiomyopathy, affecting up to 1 in 7 cats [11,12,13,14]. One study in a colony of Maine Coon cats with severe HCM, characterized by left atrial (LA) enlargement, left ventricular (LV) wall thickening and end-systolic cavity obliteration, found increased platelet activation, platelet-derived microparticles and platelet endothelial adhesion molecules compared to control cats [11]. Studies also documented platelet hyperresponsiveness to agonists in cats with HCM and transient myocardial thickening [12,13,14]. A study found a significant association between platelet priming and intracardiac thrombosis in cats with transient myocardial thickening due to wildfire-related injuries and smoke inhalation. Interestingly, despite LA enlargement, LA systolic function was increased in these cats, suggesting that underlying cardiomyopathies in cats are associated with increased platelet priming [14]. Platelet priming is a cellular response that leads to unrestrained and amplified platelet activation. However, the mechanisms underlying the observed platelet priming are not well understood.

### 2.2. Systemic Hypercoagulability

Secondary hemostasis further reinforces the initial platelet plug through the polymerization of fibrin, which is formed by a cascade of enzymatic reactions that convert prothrombin to thrombin. Systemic hypercoagulability, which is caused by an imbalance of excess thrombin generation or deficiencies in intrinsic anticoagulants, has been hypothesized to be the cause of FATE in cats with cardiomyopathies. There is, however, conflicting evidence that supports this hypothesis. One study, which assessed systemic coagulation by measuring thrombin antithrombin (TAT) complexes, antithrombin, factor VIII activity, and fibrinogen, found that a higher proportion of cats with clinical evidence of thrombosis (spontaneous echocardiographic contrast, FATE) had increased fibrinogen and TAT concentrations compared to healthy controls. However, their overall distributions were not significantly different [15]. Increased TAT complexes and decreased antithrombin concentration could indicate active thrombin generation and consumption of antithrombin [16]. In addition to plasma proteins, blood cells like platelets, erythrocytes, and leukocytes are integral to the activation of coagulation factors and fibrin, a process known as the cell-based model of coagulation [17]. Investigators have evaluated the cell-based model of coagulation using viscoelastic assays in whole blood with inconclusive results. One study, which utilized a novel point-of-care viscoelastic assay (VCM VET), found that cats with FATE were hypocoagulable as demonstrated by decreased alpha angle and maximum clot formation (MCF) when compared to healthy cats [18]. These observations were hypothesized to be secondary to consumptive thrombocytopenia, hyperlactatemia, or suboptimal blood collection. Another study that utilized the same assay in cats with FATE with and without congestive heart failure did not demonstrate any evidence of systemic hypercoagulability when compared to healthy cats [19]. However, these results should be interpreted with caution since neither study excluded cats with concurrent thrombocytopenia. Platelet count has been shown to be directly proportional to MCF on VCM VET despite clopidogrel treatments in cats [20]. Additionally, VCM has low sensitivity in detecting hypercoagulability in dogs with one study reporting a low positive predictive value for hypercoagulable sick dogs when compared to thromboelastography (TEG) [21,22]. VCM’s sensitivity and specificity in detecting feline hypercoagulability are currently unknown. While further research is needed to validate the use of viscoelastic testing in cats, the current evidence suggests that hypercoagulability alone is not the sole driving force of FATE pathogenesis.

### 2.3. Alterations in Fibrinolysis

Fibrinolysis is a highly regulated process aimed at re-establishing blood flow after clot formation via active thrombolysis. Cross-linked fibrin is cleaved by plasmin via formation of a tertiary complex consisting of tissue plasminogen activator or urokinase plasminogen activator, fibrin, and plasminogen [23]. A recent retrospective study by Busato et al. showed that the FATE prevalence is associated with the manifestations of CHF. Interestingly, cats with large volume pleural effusion were found to have a lower incidence of FATE (6.6%) compared to those with cardiogenic pulmonary edema (33%). The difference in FATE incidence is hypothesized to occur due to the intrinsic fibrinolytic properties in pleural effusion being resorbed into the systemic circulation, thus preventing thrombus formation [24]. Pulmonary edema might also be more proinflammatory than pleural effusion, leading to increased immunothrombosis, an intricate process that links systemic inflammation with coagulation.

### 2.4. Immunothrombosis

Activation of innate immunity and systemic inflammation may play a vital role in FATE pathogenesis [25,26]. As a vital component of the host defense mechanism, immunothrombosis occurs when infections or inflammation activate the immune and coagulation systems to facilitate microvascular thrombosis, preventing systemic dissemination of pathogens and promoting wound healing [27,28,29,30]. However, dysregulation of immunothrombosis has been demonstrated to cause systemic hypercoagulability and thromboembolic complications in humans and animal models [31,32]. A key component of immunothrombosis is the formation of neutrophil extracellular traps (NETs), which are decondensed extracellular chromatin decorated with antimicrobial proteins like neutrophil elastase, cathepsin G, and myeloperoxidase [33]. NET formation, also known as NETosis, plays an essential role in innate immunity by entrapping and killing microorganisms by subjecting them to high concentrations of antimicrobial proteins [25]. However, overzealous NETosis can lead to excessive clot formation, as NETs possess prothrombotic properties [34,35,36]. Circulating NETs in the form of cell-free DNA and citrullinated histones (citH) have been characterized in cats with FATE and HCM. Cell-free DNA (cfDNA) was observed to be higher in cats with FATE, but was not associated with any known risk factors of FATE [37]. Further characterization of cfDNA fragments revealed that prothrombotic cfDNA fragments that are greater than 300 base pairs were detected only in cats with HCM, with or without FATE [37]. The size profile of these cfDNA fragments indicated that an active process like NETosis, rather than a degradative process, was the primary cause for increased cfDNA levels [37]. Citrullinated histones, which are modified histones specific to NETs formation, were also significantly elevated in cats with HCM and FATE. Interestingly, the study showed that none of the clinically healthy cats had any detectable levels of citH. Additionally, it demonstrates that plasma citH is superior to cfDNA in correlating with known risk factors of thrombosis on echocardiogram [15,25]. In human and murine studies, histones are known to prime platelets to be hyper-responsive to physiologic agonists to augment adhesion, induce activation, and accelerate platelet-dependent thrombin generation via Toll-like receptors 2 and 4 [38,39]. Hence, histones may be a potential biomarker and therapeutic target for HCM cats and cats with FATE.

### 2.5. Blood Stasis

Alterations in hemodynamics within the heart occur in cats with HCM or other types of cardiomyopathies due to structural cardiac changes. In HCM, marked elevation in LV pressure caused by myocardial thickening and diastolic dysfunction leads to LA pressure overload, LA dilation, and, eventually, LA systolic dysfunction [40]. Decreased LA contractility causes blood flow stasis, facilitating blood cells, fibrinogen, and clotting factors to aggregate, forming intracardiac thrombus [40]. Spontaneous echocardiographic contrast (SEC), commonly referred to as “smoke” within the LA, is an ultrasonographic finding of increased echogenicity due to red blood cell aggregates formed at low blood flow velocity [40]. SEC and intracardiac thrombus within the LA appendage are high-risk factors for FATE development. Decreased LA blood flow, measured as left auricular flow velocity on an echocardiogram, is also an independent predictor of SEC and FATE [40]. 

### 2.6. Endothelial Injury

In healthy animals, the endothelium produces several anticoagulant molecules and vasodilatory agents to prevent excessive clot formation. These products include protein C receptors, a small proportion of protein C, thrombomodulin, prostaglandin, tissue factor pathway inhibitor, and heparan sulfate [17,41]. Once bound with thrombin, thrombomodulin (TM), an endothelial glycoprotein, exerts its anticoagulant effects by activating protein C to not only inhibit factors Va and VIIIa but also accelerate fibrinolysis. The TM-protein C system also possesses anti-inflammatory and cytoprotective properties [42]. Thrombomodulin has been demonstrated to be decreased in cats with overt HCM, indicating that endothelial dysfunction may play a role in hypercoagulability in cats with cardiomyopathies [43]. The endothelium also produces proteases, mainly tissue plasminogen activator, to induce fibrinolysis by converting plasminogen to plasmin [44]. Endothelial injuries in HCM caused by severe LA dilation and stretching of the myocardium may lead to exposure of subendothelial matrices and upregulation of procoagulant proteins. Upon endothelial injury or activation via thrombin, von Willebrand factor (vWF), a glycoprotein that resides within Weibel-Palade bodies, is secreted extracellularly. Following the cleavage of vWF by the enzyme ADAMTS13 to its less adhesive forms, vWF mediates platelet adhesion on the endothelium. In humans with HCM, vWF plasma concentrations are significantly increased secondary to microvascular dysfunction [45]. Increased circulating vWF, measured by immunodetection, has been shown to be associated in cats with acute FATE [46]. At the same time, vWF is shown to be upregulated in the endocardium of HCM-affected cats [47]. This evidence further supports the notion that cardiomyopathies can exacerbate endocardial and endothelial activation to facilitate thrombus formation.

## 3. Knowledge Gap and Future Research Directions in FATE Pathogenesis

### 3.1. Procoagulant Platelets

While increased platelet activation has been repeatedly shown to be associated with cats with cardiomyopathies, a lesser-known phenomenon is the effects of cardiomyopathies on different platelet phenotypes. In addition to the well-studied aggregatory platelets, procoagulant platelets are now a recognized sub-population of platelets, which exhibit distinct morphological and functional characteristics. Persistent exposure to potent agonists like collagen and thrombin causes platelets to lose their typical discoid shape and adopt balloon-shaped phenotypes with membrane blebbing. This transformation is accompanied by a sustained increase in intracellular calcium and the externalization of electronegative phospholipids like phosphatidylserine (PS), which enhances the assembly of coagulation factor complexes such as tenase and prothrombinase complexes to accelerate thrombin generation [48]. Through clot retraction, procoagulant platelets are translocated to the thrombus periphery, where increased fibrinogen binding further strengthens the thrombus structure [49]. While their role in hemostasis is crucial, an imbalance that favors their formation may lead to prothrombotic conditions such as ischemic stroke, coronary heart disease, deep vein thrombosis, and spontaneous intracranial hemorrhage in humans [50,51,52]. A recent study characterized the formation of procoagulant platelets in healthy cats using a set of platelet markers, including P-selectin, inner mitochondrial membrane potential (Δψm), and PS [53]. The results showed that, similar to human platelets, stimulation of feline platelets with thrombin and glycoprotein VI agonists induces similar phenotypes such as PS externalization, P-selectin expression, and a loss of Δψm. Future research should focus on the effects of cardiomyopathies on this dichotomy of platelet activation, since current antiplatelet strategies do not target the formation of procoagulant platelets.

### 3.2. Knowledge Gaps in Immunothrombosis

In addition to increased circulating citH and cfDNA in cats with HCM, arterial thrombi in cats are rich in citH, which suggests that NETs play an active role in the pathogenesis of FATE [54]. The underlying mechanisms of increased NETosis, however, are not well understood in cats with cardiomyopathies. One potential mechanism is that increased platelet activation may facilitate platelet-neutrophil interactions to promote NETs production. Activated platelets have been shown to stimulate neutrophils to produce NETs by directly or indirectly binding to neutrophils. Cellular mechanisms mediating this cell-to-cell crosstalk are species-dependent. For example, while the binding of platelet P-selectin to the neutrophil receptor, P-selectin glycoprotein ligand-1 (PSGL1), is crucial for NETosis in murine sepsis models, human neutrophils, on the other hand, require firm adhesion mediated via neutrophil integrins and platelet glycoprotein 1bα [55,56,57]. Increased circulating citH due to NETosis may further exacerbate platelet activation in cats with cardiomyopathies. Activated platelets also release a number of soluble mediators, creating an environment that favors the production of NETs by nearby neutrophils, creating a vicious cycle. Future research should focus on uncovering these underlying mechanisms as they are instrumental in the discovery of new therapeutic targets. Research is ongoing to explore and target the immunothrombotic interactions between neutrophils and platelets to reduce inflammation, NET production, and platelet priming.

### 3.3. Congestive Heart Failure and Fibrinolysis

Lastly, the effects of different manifestations of CHF on fibrinolysis require further investigation. Because cats at risk of FATE often have concurrent CHF, this has direct implications for optimizing the primary prevention of FATE. No studies have specifically investigated the fibrinolytic and coagulation profiles in cats with different CHF manifestations. One study described a trend of decreased plasminogen activity in cats with acquired heart disease, while others have found evidence of hypercoagulability, hyperfibrinogenemia, and increased blood viscosity in cats with HCM hypertrophic cardiomyopathy [15,58,59]. However, no studies, to date, have directly compared fibrinolysis in cats with CHF manifesting as large-volume pleural effusion or pulmonary edema. The biggest challenge of quantifying fibrinolysis is that the overall process is dependent on the quantity and quality of various fibrinolytic enzymes like tissue plasminogen activator and their inhibitors like plasminogen activator inhibitor-1 (PAI-1) and thrombin-activable fibrinolysis inhibitor (TAFI). Biomarkers of fibrinolysis, while clinically accessible, may be limited in their diagnostic ability in evaluating fibrinolysis. Fibrin degradation products, including D-dimer, a fragment of cross-linked fibrin, are associated with increased fibrinolysis due to recent or ongoing clot formation [60]. There is an association between plasma levels of D-dimers and FDPs for human patients with suspected thromboembolic disease [61]. A study concluded that elevated D-dimer concentrations were found in HCM cats with FATE, as well as cats with evidence of systolic anterior motion of the mitral valve [62]. D-dimer, however, has poor sensitivity and specificity in predicting thrombosis in cats with HCM. A study of asymptomatic HCM cats did not demonstrate any association between D-dimer levels and LA size when compared to healthy cats [8]. Another potential fibrinolysis biomarker is plasmin-alpha-2 antiplasmin complex (PAP). Alpha-2 antiplasmin inhibits plasmin by forming a covalent complex and is an indication of fibrinolysis activation. In humans, PAP is weakly associated with an increased risk of venous thromboembolism and ischemic stroke, and is increased in patients with acute myocardial infarction [63,64,65,66]. Its use has not been validated in cats.

Clot lysis assays have been used in human medicine as a standard method to evaluate overall fibrinolytic potential over time, but this has not been validated in cats. Assessment of fibrinolysis in whole blood using the viscoelastic assay, VCM VET, in cats with CHF has failed to demonstrate any differences in clot degradation between FATE cats with or without CHF. However, the addition of exogenous fibrinolysis activators, like tissue plasminogen activator, may increase the sensitivity of fibrinolysis potential, as it may test for the function of modulatory molecules such as PAI-1 and TAFI.

### 3.4. Blood Flow Stasis and Endothelial Injury

While it is universally accepted that SEC is associated with severe LA dysfunction, the blood elements causing the characteristic swirling pattern of blood flow on echocardiogram in cats are unclear. Previous in vitro studies in human whole blood have shown that blood echogenicity is largely dependent on the formation of erythrocyte-fibrinogen aggregates under low shear forces [67,68]. Although platelets are not echogenic in nature, platelet aggregates were found to be echogenic once they are activated. This was further confirmed by Zotz et al., who demonstrated that blood collected from the LA of human patients with SEC had significantly higher numbers of activated platelets, leukocytes, and platelet–monocyte aggregates [69]. Further studies in cats are needed to evaluate if platelets and leukocytes are involved in SEC formation.

Recall that vWFs are cleaved rapidly from their largest high-molecular-weight multimers to their less adhesive forms once they are released into the circulation. This proteolytic transformation of vWF is largely dependent on shear stress. For that reason, different cardiomyopathy phenotypes may influence the functional capacity of vWF in cats. In humans with the obstructive form of HCM, individuals with peak left ventricular outflow tract gradient of >30 mmHg are found to have functional impairment of vWF, characterized by decreased collagen-binding activity and lower levels of high molecular weight multimers [70]. These findings suggest that the thrombotic risk in cats with cardiomyopathies, especially HCM, may vary based on the degree of left ventricular outflow tract obstruction, and further studies are needed to better characterize the acquired changes in vWF structure and function. On the other hand, low shear stress in severely dilated LA with poor systolic function may increase the thrombogenic properties of vWF. In a study of human patients with atrial fibrillation, vWF quantity and function are directly proportional to LA function and are independent predictors of intracardiac thrombosis [71].

## 4. Current Risk Factors of FATE

Early identification of risk factors requires sensitive and specific diagnostic tests to identify risk factors associated with the development and progression of FATE. To date, the only reliable diagnostic test shown to predict SEC and FATE is echocardiography [8]. Table 1 summarizes the reported risk factors of FATE in cats.

### 4.1. Echocardiography

**Figure 3:**
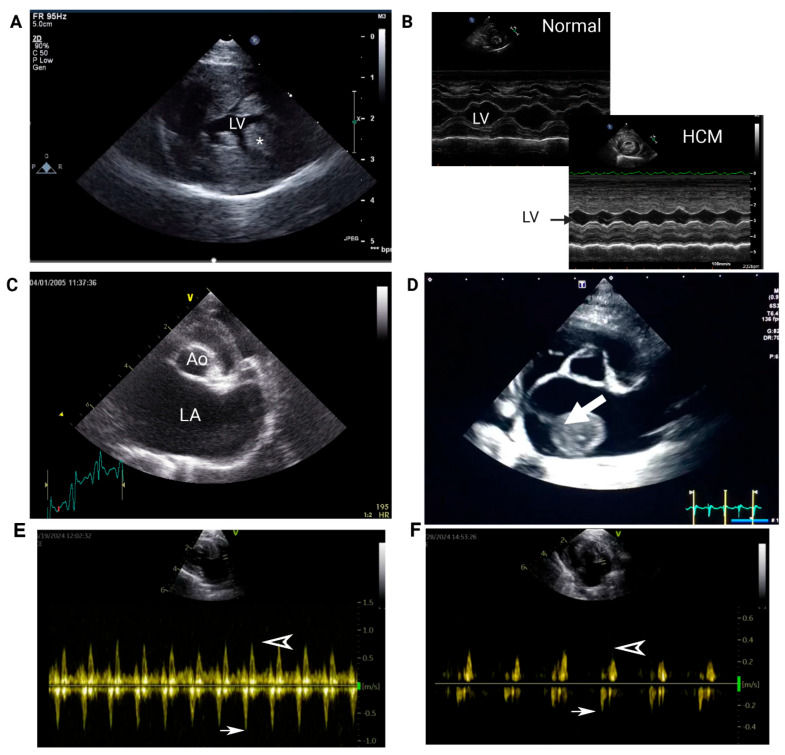
Representative images of echocardiograms in cats with hypertrophic cardiomyopathy. (**A**) Right parasternal short-axis view of the left ventricle (LV). Note the marked thickening of the LV free wall (* indicates the papillary muscle). (**B**) M-mode of the LV at the level of the papillary muscle in a normal cat and a cat with HCM. Note the significant reduction in LV diameter during diastole. (**C**) Right parasternal short-axis view at the level of the left atrium (LA) demonstrating marked LA enlargement (Ao = aorta). (**D**) An organized thrombus (arrow) is visible in the LA with marked chamber enlargement in an asymptomatic cat with HCM. (**E**) Pulsed Doppler tracing from a cat without heart disease. Normal left auricular flow velocities showing mean emptying flow velocity of 0.8 m/s (arrow). The filling flow velocity is shown as an arrowhead. (**F**) Recorded pulsed Doppler tracing from a cat with HCM and left atrial enlargement showing low mean flow velocity (arrow).

On echocardiogram, the presence of LA enlargement, commonly assessed by measuring the ratio of the diastolic LA diameter to aortic root diameter from a right-sided short axis view (LA:Ao, Figure 3C), is a significant risk factor for cardiac-related deaths such as CHF and FATE [40,72,73]. With proper training, skilled practitioners can acquire essential views, such as those shown in Figure 3, on point-of-care ultrasound to assess LV thickness and LA size in cats. A severely enlarged LA is an indication of concurrent or imminent congestive heart failure and often signals LA dysfunction. Systolic dysfunction of LA, evaluated by measuring the fractional shortening of LA or LA appendage flow velocities, increases the risk of blood flow stasis, causing SEC or intracardiac thrombosis (Figure 3D). LA appendage flow velocity <20 cm/s on echocardiography indicates severe LA systolic dysfunction and is an independent predictor of SEC formation and, possibly, FATE (Figure 3E,F) [40]. Previous thromboembolic events and reduced LA fractional shortening are also prognostic indicators for FATE and cardiac-related mortality [72,73].

### 4.2. Cardiac Biomarkers

Currently, there are no clinically available biomarkers that can accurately predict the onset of FATE. N-terminal prohormone of brain natriuretic peptide (NT-proBNP) is a cleaved by-product of B-type natriuretic peptide synthesized in the cardiac myocytes in response to stretch or stress. In an emergency setting, NT-proBNP is most useful when it is used in cats with respiratory distress to differentiate between cardiogenic and respiratory causes. If access to a cardiologist or point-of-care (POC) ultrasound is not possible, it is reasonable to measure serum or plasma NT-proBNP, especially in cats with radiographic evidence of cardiomegaly [74,75]. It is important to note that NT-proBNP is moderately accurate for detecting advanced stages of HCM but may be normal in cats with mild subclinical HCM [74]. Studies evaluating the use of POC NT-proBNP in apparently healthy cats showed that NT-proBNP testing is specific (96% to 100%) with an acceptable range of positive predictive values (78% to 100%) [76,77]. This means that a cat with a positive test likely has underlying heart disease and should be referred for echocardiography. The positive predictive value, which is an indication of true positives, would theoretically be higher in a population of cats greater than 7 years of age, given the higher prevalence of heart disease in geriatric cats. However, it is important to recognize that the cutoff of 200 pmol/L on POC NT-proBNP may result in false negatives, as cats with asymptomatic HCM can be found in the 100 to 200 pmol/L range [77].

Elevated cardiac troponin I (>0.06 ng/mL) showed high sensitivity and specificity for asymptomatic HCM cats compared to healthy cats [78,79]. However, comorbidities such as kidney disease, hyperthyroidism, and hypertension can cause elevated troponin I [78]. A study found that normal troponin I levels were detected in asymptomatic HCM cats without LA dilatation [79]. One study evaluating troponin I in feline HCM noted that two of the cats with thromboembolic disease had the highest elevations of the study population [80]. Other studies have shown a significant elevation in troponin I for cats with acute FATE compared to cats with mild to moderate HCM [75,78]. One cat within the study had a severely elevated troponin I six hours prior to the onset of FATE [75]. Thus, further studies are required to determine the predictive role of this biomarker. The authors recommend that cardiac biomarkers should be utilized in conjunction with other diagnostic tests. Research is ongoing to develop reliable diagnostic tests that may be readily available for veterinary practitioners to identify cats at risk of FATE.

## 5. Knowledge Gaps and Future Directions in Identifying Risk Factors

### 5.1. Platelet Heterogeneity as a Risk Factor of FATE

Circulating platelets in cats are highly variable in size, and increased mean platelet volume (MPV) in cats with FATE may indicate platelet activation due to micro-aggregation or underlying inflammation [81]. In people with heart diseases, elevated MPV is associated with increased hospitalization. However, its use in risk stratification in cats with cardiomyopathy requires further investigation. In recent years, researchers have increasingly recognized the importance of platelet heterogeneity, which explains why platelets behave and function differently between individuals. Assessing the proteome and RNA expression in platelets from cats with cardiomyopathies would be an important first step in characterizing platelet heterogeneity and its association with FATE. Lastly, the diagnostic significance of procoagulant platelets as biomarkers to predict thrombotic events in cardiomyopathic cats should be evaluated.

### 5.2. Hematological Variables as Risk Factors of FATE

Neutrophil-to-lymphocyte ratio (NLR) is an indicator of acute inflammatory stress response and an imbalance between innate and adaptive immunity during systemic diseases. Normal NLR ranges from 1 to 2, and an elevated NLR (>2) is associated with neoplasia and inflammatory disorders in cats [82,83,84]. Increased NLR has been shown to be a negative prognostic indicator in cats with HCM [82]. According to one retrospective observational study, increased NLR >4.46 was associated with reduced median survival time and cardiac-related deaths in cats with ACVIM Stages B and C cardiomyopathies, including those with intracardiac thrombus and spontaneous echo contrast [82]. Further research is needed to confirm whether serial assessment of NLR or NLR trends could be a more robust biomarker for assessing thrombotic risk in cardiomyopathic cats.

### 5.3. NETs as Biomarkers of FATE

Increased circulating NETs in the form of cell-free DNA and citH have been documented in cats with HCM. Of the two biomarkers, citH is significantly correlated with echocardiographic risk factors, including LA enlargement and left auricular flow velocity, suggesting its potential as a diagnostic marker for predicting FATE. Due to the cumbersome nature of electrophoresis and Western blot analysis, future research should be directed towards developing a feline-specific point-of-care assay that is highly sensitive and specific in measuring plasma citH. In addition, a longitudinal study that evaluates plasma citH over time in cats with subclinical HCM would provide additional diagnostic information on the pathophysiologic significance of immunothrombosis [25,54].

### 5.4. Other Biomarkers of FATE

MicroRNAs are non-coding RNAs that either silence or degrade messenger RNA sequences to regulate gene expression. Cats with HCM were previously found to have distinct microRNA expression profiles for LV and LA when compared to healthy cats without HCM [85]. In humans, circulating microRNA profiles have been evaluated in individuals using an unbiased array approach to distinguish not only those with clinical HCM, but also subclinical HCM from healthy controls [86]. These findings highlight the potential of utilizing circulating microRNAs for identifying cats with subclinical HCM, allowing for early thromboprophylaxis to prevent FATE.

## 6. Current Recommendations of Primary and Secondary FATE Prevention

Current guidelines recommend that all cats in Stage B2, C, and D cardiomyopathies should be treated with clopidogrel as primary thromboprophylaxis [87]. This recommendation is largely based on the aforementioned ex vivo studies as well as 2 prospective clinical trials. The FATCAT study showed that cats that were randomized to receive clopidogrel after an initial FATE event had a longer median time to FATE recurrence and a longer median survival time compared to those randomized to aspirin treatment [6]. There is currently not enough evidence to recommend the sole use of direct oral factor Xa inhibitors like rivaroxaban or apixaban, or anticoagulants like unfractionated heparin therapy and lower molecular weight heparin [87]. The SUPERCAT study, which compared clopidogrel to once-daily rivaroxaban treatment in cats that survived their first event of FATE, demonstrated no difference in ATE recurrence between the two groups [88]. However, this study was likely underpowered, and its results may have been biased by the greater progression of cardiomyopathy in cats within the rivaroxaban group compared to the clopidogrel group. In another ex vivo study, healthy cats treated with rivaroxaban alone had evidence of platelet priming compared to those treated with clopidogrel alone or dual agent therapy with clopidogrel and rivaroxaban [89]. The underlying mechanisms behind these findings remain unclear, necessitating further studies to determine whether anti-platelet therapy may have a cardioprotective effect. Additionally, it is important to note that the current evidence may not directly apply to the primary prevention of FATE, as major clinical trials have only included cats that survived an initial episode of FATE, which may have introduced selection bias and overstate the survival benefit of antithrombotics.

Dual agent therapy (DAT) aims to simultaneously target the interactions between primary and secondary hemostasis to further dampen platelet activation and thrombin propagation. A retrospective study observing the use of clopidogrel and the activated factor X (FXa) inhibitor, rivaroxaban, found favorable outcomes in clinical cats receiving DAT with 0% incidence of FATE in high-risk cats and a recurrence rate of 16.7% [5]. Another retrospective study that evaluated the use of continuous infusion of enoxaparin intravenously with clopidogrel in 36 FATE cases showed that enoxaparin administration was safe, but the in-hospital mortality rate remains high at 48%, which is similar to findings from other studies. [90]. An ex vivo study in a colony of healthy cats found that DAT with rivaroxaban and clopidogrel not only decreased circulating activated platelets but had synergistic inhibitory effects on thrombin-mediated platelet activation and platelet-dependent thrombin generation compared to rivaroxaban or clopidogrel alone. Further clinical trials are needed to evaluate the benefit of DAT in reducing the incidence of FATE in cats with clinical HCM.

## 7. Knowledge Gap and Future Directions in Optimizing Prevention Strategies

### 7.1. Genetic Testing

Clopidogrel is an antiplatelet drug that irreversibly inhibits the ADP receptor, P2Y_12_, after it is metabolized to its active metabolite by the liver [91]. The pharmacodynamic response to clopidogrel in cats is highly variable, with reduced efficacy reported in up to 30% of cats [12,45]. This, otherwise known as “clopidogrel resistance”, is caused by a single-nucleotide polymorphism within the ADP receptor gene, *P2RY1*, which encodes the ADP receptor, P2Y1 [92]. The P2RY1:A236G variant was found to be associated with a significant reduction of platelet inhibition by clopidogrel in HCM cats [92]. The exact mechanism of this altered pharmacogenomic response is unknown, since the clopidogrel active metabolite inhibits the other ADP receptor, P2Y12, and not P2Y1. Peptide modeling suggests that variants in *P2RY1* may cause either upregulation or gain-of-function of the P2Y1 receptor. Clopidogrel may, therefore, not be the optimal antithrombotic therapy in cats with the heterozygous or homozygous variant. Given the high prevalence of clopidogrel resistance (homozygous 16.3%, heterozygous 51%), precision medicine utilizing molecular testing for this mutation may be a sensible approach to evaluate clopidogrel response and optimize thromboprophylaxis in cats with HCM [92]. Historically, the limited availability of genetic testing has been a barrier to optimizing antiplatelet therapy through a precision medicine approach. However, with the recent availability of feline-specific P2RY1 testing and increasing access to commercial whole-genome sequencing, clinicians in general practice now have tools to individualize antithrombotic therapy. This also underscores the importance of future clinical trials evaluating the clinical outcomes in cats with P2RY1-guided antithrombotic therapy in at-risk cats.

Further research in determining the microRNA profiles of cats with HCM may aid in distinguishing cats with subclinical disease and identifying those at increased risk for developing FATE [93]. How to effectively apply microRNA profiling in clinical practice remains unclear.

### 7.2. Platelet Function Testing in Clinical Practice

Light transmission aggregometry is the gold standard of monitoring antiplatelet therapy, but is poorly standardized among laboratories. Because it requires operators to generate platelet-rich plasma, in vitro activation during the centrifugation process can severely impact the utility of the assay in a clinical setting. In addition, the diagnosis of clopidogrel resistance requires aggregation measurements before and after treatments, making it impractical to utilize in practice. A more user-friendly and automated version of platelet aggregometry utilizing whole blood samples has successfully been validated in cats to evaluate clopidogrel response, but its availability is restricted to research or referral institutions [92,94]. The Platelet Function Analyzer (PFA-100 or 200) measures the duration required for the formation of the platelet plug within the aperture under high shear rates. Despite its ease of use, flow obstruction caused by in vitro platelet activation and aggregation may present a problem for assessing platelet function in cats [95,96]. PFA, therefore, may be a suitable test in assessing the antiplatelet effects of clopidogrel, although its diagnostic ability in identifying cats with clopidogrel resistance requires further evaluation [95,97]. Another POC assay called Plateletworks is an accessible test as it measures platelet aggregation by comparing platelet counts in EDTA-anticoagulated blood and specialized blood tubes containing agonists like ADP or collagen. Blood tubes containing platelet agonists induce platelet activation and aggregation, which then lowers the platelet count. The main advantage of this assay is that no specialized equipment is needed. Aggregation is mathematically derived by comparing platelet counts in blood tubes with or without platelet agonists using an automated analyzer. While the assay shows some promise in assessing antiplatelet effects of clopidogrel, it may not be sensitive enough to detect the drug effect of aspirin [98,99].

### 7.3. Monitoring of Anticoagulants 

Current guidelines do not have specific recommendations on the routine monitoring of anticoagulants like low molecular weight heparins (LMWH) or direct oral FXa inhibitors. The gold standard assay for assessing the efficacy of LMWH and direct oral anticoagulants is measuring anti-factor Xa activity (aXa). In humans, the recommended therapeutic target of LMWH is 0.5 to 1 IU/mL [100]. However, the optimal ranges of aXa that would offer the best protection have yet to be established in cats. Compared to humans and dogs, cats have more rapid elimination of LMWH, while rivaroxaban has more predictable pharmacodynamic effects and a longer median half-life of about 8 h [101,102]. In one ex vivo study in cats, rivaroxaban achieved the recommended human target range for thromboprophylaxis, and the duration of protective aXa was dose-dependent [101]. Based on this data, it is difficult to justify routine testing of aXa activity to evaluate the anticoagulant effects of rivaroxaban in cats. Because the elimination of rivaroxaban is largely dependent on renal function and, to some degree, hepatic function, human beings with hepatic or kidney diseases receiving rivaroxaban are at a higher risk of developing hemorrhagic complications. For that reason, aXa testing is reserved for those with decreased renal function. Consequently, assessing rivaroxaban-mediated aXa in cats with kidney disease or those who develop SEC or intracardiac thrombosis during treatment is a sensible approach to guide dose adjustments. Future research should explore aXa-guided dose adjustments of direct oral anticoagulants to prevent FATE and its recurrence.

### 7.4. Viscoelastic Testing to Monitor Antithrombotic Therapies

Point-of-care viscoelastic testing provides a real-time quantitative and qualitative assessment of global coagulation, including primary and secondary hemostasis, contribution of blood cells to clot formation, and fibrinolysis [103]. Several analyzers are currently available to veterinary practitioners, including TEG, viscoelastic monitor (VCM-VET), and rotational thromboelastometry (ROTEM). Viscoelastic testing may be a valuable tool in guiding antithrombotic treatments in cats with FATE, but many questions remain due to the high interassay and interindividual variabilities and the lack of consensus on how to define hypercoagulability on viscoelastic testing assays in HCM-affected cats. Due to the lack of standardized reference intervals and considerable intraassay variability, further research to utilize viscoelastic testing for serial monitoring or assessing therapeutic response may be more beneficial than as a standalone diagnostic tool. One study evaluated TEG and VCM-VET for monitoring clopidogrel treatment in healthy cats. Using light transmission aggregometry as a gold standard, investigators found that cats that were resistant to clopidogrel had decreased R time on TEG, which suggest that R time may be used as a diagnostic tool to assess clopidogrel response [20]. Because viscoelastic tests assess both clot formation and breakdown, treatment decisions, whether anticoagulant or antiplatelet therapy, can be based on the baseline values that indicate a hypercoagulable state. For example, hyperreactive platelets are associated with increased maximum amplitude on TEG in a human study [104]. Since there is an identified association between platelets and intracardiac thrombosis in cats, elevated MA in FATE cats could encourage research in the safety and efficacy of alternative antiplatelet drugs in veterinary patients, beyond clopidogrel and aspirin. Recent studies have evaluated the utilization of VCM-VET in cats with HCM and FATE, given that it only requires a small volume of blood, and no specialized equipment or reagents are needed. A study evaluated viscoelastic testing to assess platelet function monitoring for healthy cats receiving clopidogrel and demonstrated hypercoagulable variables such as shortened K time and increased alpha angle on TEG and increased maximum clot formation (MCF) on VCM-VET [20]. Table 2 summarizes the utility of each viscoelastic test. More studies are required to identify the associations between viscoelastic parameters and clinical thrombotic risk in cats.

## 8. Novel Antithrombotic Therapies

### 8.1. Rapamycin

Rapamycin, or sirolimus, is an inhibitor of the mammalian/mechanistic target of rapamycin (mTOR) pathway, which plays an important role in cell growth, metabolism, and immune responses. Rapamycin has gained significant interest for its therapeutic potential in cardiovascular diseases due to its ability to modulate key cellular processes such as inflammation and proliferation [105]. Delayed-release rapamycin, which was recently approved by the U.S Food and Drug Administration for use in cats with HCM, has cardioprotective effects by either halting or reversing concentric hypertrophy of the myocardium [106]. A multi-omic study also revealed that cats treated with rapamycin had a downregulation of proteins associated with complement activation, humoral immunity, and inflammatory response, indicating a potential modulating effect on inflammation and immunothrombosis [107]. An in vitro study in human platelets demonstrated that rapamycin reduces procoagulant platelet formation by protecting mitochondrial membrane potential (ΔΨm) and limiting phosphatidylserine (PS) externalization to prevent excessive thrombin generation via an mTORC1-independent mechanism [108]. However, the effects of rapamycin on other platelet functions remain poorly understood, with most studies limited to in vitro models, which yield conflicting results. In an in vitro study, rapamycin increases platelet aggregation and secretion in response to ADP and thrombin, while another study showed modulation in collagen response by reducing platelet aggregation and spreading [109,110]. In cats, a recent ex vivo study revealed that low-dose rapamycin administered orally every 7 days for 4 weeks significantly diminished the procoagulant potential of platelets by preventing the loss of ΔΨm. Additionally, rapamycin also modulated ADP-induced platelet activation [111]. While rapamycin holds promise for managing various cardiovascular conditions, further research is necessary to fully understand its long-term safety and potential to be used synergistically with existing therapies.

### 8.2. Non-Anticoagulated Heparins

The anticoagulant activity of UFH can be considered an undesirable effect, especially in cats with unknown coagulation status. While the use of unfractionated heparins (UFH) as an anticoagulant in cats with FATE is not novel, there is a knowledge gap concerning the use of UFH as an anti-inflammatory and anti-immunothrombotic agent to be utilized as a primary prevention therapy. Heparin’s anti-inflammatory properties stem from its ability to interact with proteins such as complements and histones, which are responsible for mediating systemic inflammatory response [112]. Taking that into consideration, there is ongoing research investigating new heparin derivatives, known as non-anticoagulated heparins (NAH), with preserved anti-inflammatory properties and reduced or obliterated anticoagulant activities. Currently, there are multiple formulations of NAH, with most being desulfated heparin since the sulfated nature of heparin confers a negative charge, allowing it to interact with multiple proteins, such as antithrombin (9). The downside of desulfated heparin is the variable effects it has on histone inhibition; however, other studies indicated that selectively desulfated heparin retains a high degree of histone scavenging capability. Considering that elevated histones in cats with HCM may play a role in CATE pathogenesis, more research is needed to evaluate the efficacy and safety of NAH in cats as a primary and secondary thromboprophylaxis.

### 8.3. Thrombolytic Therapy

Tissue plasminogen activator (tPA) is a thrombolytic agent that works by converting plasminogen to plasmin, which specifically degrades cross-linked fibrin in blood clots. It is considered the standard of care for treating acute ischemic stroke and myocardial infarction in humans since tPA targets thrombus more selectively than streptokinase [113,114]. The lack of high-quality evidence in veterinary studies limits the widespread use of thrombolytics and, therefore, should be used with caution in cats with FATE. Given that intracranial hemorrhage has been reported in approximately 2% of human patients treated with reteplase and alteplase, yet no hemorrhagic events were observed in cats enrolled in the BLASTT study, it is plausible that species-specific differences exist [115,116]. These findings highlight the need for further investigation into feline-specific dosing regimens to optimize the risk-benefit profile of thrombolytic therapy in this population. While the CURATIVE guidelines suggest that thrombolysis may be considered in cats with acute FATE, the ACVIM consensus statement advises against thrombolysis treatment for FATE [91,117]. To date, the best evidence for thrombolytic therapy comes from the BLASTT (Bilateral Lysis of Aortic Saddle Thrombus with Early Tissue Plasminogen Activator) study, a randomized, prospective clinical trial that compared alteplase (a second-generation tPA) to placebo. Similar to human recommendations, enrollment of cats was restricted to those that received tPA or placebo within 6 h of a FATE event. The study found that cats that survived to 48 h of hospitalization had improved limb functions compared to the placebo group, but there were no statistical differences in survival to discharge or adverse events such as acute kidney injury and reperfusion injury [116]. The study, however, was underpowered to evaluate survival and secondary outcomes. Another thrombolytic agent, streptokinase, which non-specifically activates plasminogen to induce a systemic fibrinolytic state, was evaluated in a retrospective study of 46 cats. The study found that 24% of cats experienced clinical bleeding and prolonged coagulation times [118]. Furthermore, a prospective study of eight cats treated with streptokinase showed that none of the cats survived to hospital discharge [119]. The International Study of Infarct Survival-3 found no difference in mortality between streptokinase and tPA in human patients with an acute onset of myocardial infarction [120]. However, the tPA group had significantly more strokes suspected to be secondary to cerebral hemorrhage [120]. Another human study compared varying regimens of tPA and streptokinase with subcutaneous and intravenous heparin and concluded that accelerated tPA with intravenous heparin was superior and had a significant reduction in mortality [121]. Accelerated tPA was administered as a bolus of 0.75 mg/kg over 30 min and then 0.5 mg/kg over 60 min [121]. Urokinase, which is a direct plasminogen activator that acts systemically, was evaluated in a retrospective study involving 12 cats. The study found that 5 of the 12 cats (42%) survived to discharge while hyperkalemia developed in 25% of cases [122].

Additionally, reteplase, a third-generation tissue plasminogen activator (tPA) approved for human acute myocardial infarction, was administered to twelve cats with bilateral arterial thromboembolism, resulting in a high 75% survival rate [123]. In human medicine, the RAISE trial, which compared reteplase to alteplase in patients with acute ischemic stroke, found that while reteplase was associated with better functional outcomes, it also had a higher incidence of intracranial hemorrhage [124]. Mortality rates between the two groups did not differ significantly. These findings suggest that reteplase may offer a promising option for FATE treatment, but further research is needed to assess its safety and efficacy in veterinary patients.

### 8.4. Interventional and Surgical Thrombectomy

Due to the high mortality and poor prognosis associated with FATE, novel therapies are being explored to improve patient outcomes. One case report highlighted the success of surgical embolectomy, where a cat exhibited no clinical signs 18 months after the procedure [125]. Because FATE commonly presents with concurrent congestive heart failure and severe cardiomyopathies, medical management of FATE remains the mainstay of therapy, given the high risk of anesthesia-related complications. In addition to patient stability, additional perioperative factors such as thrombus accessibility, vessel diameter, limb viability, and time to intervention may determine the feasibility of interventions and the type of interventions [126]. To date, only case reports and case series have described the efficacy of surgical thrombectomy and rheolytic thrombectomy for the treatment of FATE [125,127]. Rheolytic thrombectomy, a procedure utilizing pressurized saline jets to lyse the thrombus in combination with vacuum-assisted aspiration of clot fragments, was performed in six cats with bilateral FATE [127]. Although successful clot dissolution was achieved in five of the six cases, only three cats (50%) survived to hospital discharge, which is similar to the 48 h survival rate in recent studies consisting of only medical management. Recurrence rate among survivors was reported to be high, with one cat with documented recurrence of FATE, while the third cat survived for two years post-intervention before succumbing to congestive heart failure and chronic renal disease.

### 8.5. Histone Deacetylase Inhibitors and Scavenging

Given the prothrombotic and proinflammatory properties of histones, future preventive strategies targeting immunothrombosis should focus on inhibiting histone modification, scavenging free circulating histones, and attenuating NETosis. While currently approved by the FDA for treating neoplastic diseases, HDAC inhibitors are also being explored for their potential therapeutic benefits in human cardiac conditions [128,129]. Overexpression of certain histone deacetylases (HDACs), which are enzymes that remove acetyl groups from histone proteins, has been linked to cardiac hypertrophy in humans [129]. Increased histone deacetylation by HDAC can augment inflammation by influencing gene expression. In mouse models, HDAC inhibitors have demonstrated cardioprotective effects. HDAC inhibitors may also decrease NET formation and subsequent release of histones, since histone acetylation can increase NET formation. Although HDAC inhibitors have been studied in canine cancer cell lines, additional research is needed to evaluate their potential benefits in FATE-affected cats [130,131]. Targeting NET formation by inhibiting histone citrullination or interfering with platelet–neutrophil interactions may help to alleviate systemic hypercoagulability. Monoclonal antibodies can be a viable therapy to target adhesion molecules that mediate the interactions between platelets and neutrophils. Lastly, besides NAH, other proteins such as Clusterin have shown promise as a histone scavenging molecule to reduce immunothrombosis in mouse models [132].

## 9. Conclusions

Research is ongoing to optimize FATE recognition and identify risk factors for FATE so that proper treatments can be administered. Currently, identification of FATE risk factors relies on echocardiographic findings, but research is underway for other biomarkers and assays to predict FATE. Antiplatelet drugs remain the cornerstone of FATE prevention and treatment. Platelet function monitoring, genetic testing, and possibly viscoelastic testing in the future can be used to assess clopidogrel resistance in cats that are on antiplatelet drugs and to aid in more directed and targeted therapies. 

## Figures and Tables

**Table 1:** Risk factors associated with cardiogenic arterial thromboembolism.

History/Clinical Assessment	Male
	Breed disposition (Ragdolls, Maine Coon) with mutation of myosin binding protein C gene (MYBPC3)
	Previous thrombotic event(s)
	Heart murmur
	Gallop rhythm
Biomarkers	Elevated troponin (>0.06 ng/mL) *
	Elevated NT-proBNP (>99 pmol/L) or POC positive (>200 pmol/L) *
Thoracic Radiographs	Cardiomegaly (VHS > 8)
	LA enlargement
Echocardiogram	LV systolic dysfunction (LV fractional shortening and emptying fraction)
	LA systolic dysfunction (Low LAA velocity, Low LA fractional shortening)
	Spontaneous echocardiographic contrast and/or intracardiac thrombus
	LA enlargement (LA:Ao > 1.6)
Electrocardiogram	Arrhythmias: atrial fibrillation, ventricular arrhythmias

* No direct association with thrombosis but likely indicates significant heart disease that warrants further investigations; LA—left atrial; LAA—left atrial appendage; LA:Ao—Left atrium to aortic root ratio; LV—left ventricular; VHS—vertebral heart score.

**Table 2:** Comparison of viscoelastic tests.

Viscoelastic Test	Results Interpretation	Pros	Cons	Sample Required
TEG	Hypercoagulability may be diagnosed by 2 or more of the following: ○ Decreased R-time ○ Decreased K time ○ Increased MA ○ Increased alpha angle ○ Increased G value Hyperfibrinolysis Increased LY30 and LY60 (>15.2%)	Global assessment of coagulation, including fibrinolysis	Influenced by hematocrit and viscosity No clinical studies evaluating the association with thrombotic risk High intra-assay variability Limited availability	Citrated or fresh whole blood
ROTEM	Hypercoagulability may be diagnosed by 2 or more of the following: ○ Decreased CT ○ Decreased CFT ○ Increased alpha angle ○ Increased MCF Hyperfibrinolysis ○ Lysis > 15% within 30–60 min ○ ML > 15% ○ Decreased LOT	Global assessment of coagulation, including fibrinolysis Point of care Separate assays to assess intrinsic, extrinsic, and fibrinogen activity	Influenced by hematocrit and viscosity No clinical studies evaluating the association with thrombotic risk High intra-assay variability	Citrated whole blood
VCM	Hypercoagulable Decreased CT and CFT Increased alpha angle and MCF Hyperfibrinolysis Increased Li30, Li45 (%)	Global assessment of coagulation, including fibrinolysis Point of care More accessible minimal training required	Readings can be affected by vibrations and movement No clinical association with thrombotic risk	Fresh whole blood

TEG—Thromboelastography; R-time—Reaction time; MA—Maximum Amplitude; LY30—Lysis at 30 minutes; LY60—Lysis at 60 minutes; ROTEM—Rotational Thromboelastometry; CT—Clotting time; CFT—Clot formation time; MCF—Maximum clot firmness; ML—Maximum lysis; LOT—Lysis onset time; VCM—Viscoelastic monitoring; Li30—Clot Lysis after 30 min; Li45—Clot Lysis after 45 min.

## Data Availability

Not applicable.
